# Beliefs About Creativity Influence Creative Performance: The Mediation Effects of Flexibility and Positive Affect

**DOI:** 10.3389/fpsyg.2018.01810

**Published:** 2018-09-24

**Authors:** Nujaree Intasao, Ning Hao

**Affiliations:** School of Psychology and Cognitive Science, East China Normal University, Shanghai, China

**Keywords:** fixed mindset, growth mindset, self-efficacy, beliefs about creativity, creativity

## Abstract

This research explores potential factors that may influence the relationship between beliefs about creativity and creative performance. In Study 1, participants (*N* = 248) recruited from upper secondary schools in Thailand were asked to solve the Alternative Uses Task (a typical divergent thinking task) and complete a series of questionnaires concerning individual beliefs about creativity and potential factors of interest. The results of structural equation modeling reveal a mediation effect of flexibility on the relationship between self-efficacy and originality. The path from self-efficacy to flexibility was also partially mediated by positive affect. Self-efficacy was also positively correlated with task enjoyment and effort. Additionally, the growth mindset was positively associated with positive affect, while the fixed mindset was positively related to negative affect. In Study 2, participants (*N* = 214) were asked to solve the Insight Problems Task (a typical convergent thinking task). The results indicate that the growth mindset was positively related to task enjoyment, effort, and positive affect. The fixed mindset was negatively related to task enjoyment, effort, and creative performance. A positive relationship between the fixed mindset and negative affect was also observed. Taken together, these findings unveil some potential factors that mediate the relationships between beliefs about creativity and creative performance, which may be specific to divergent thinking tasks.

## Introduction

### Creativity and Beliefs About Creativity

Psychologists agree upon the definition of creativity as the ability to produce work that is novel (original and unique) and useful (Stein, 1953; Sternberg and Lubart, 1993; Runco and Jaeger, 2012). From a cognitive perspective, creativity is concerned with two types of thinking, namely divergent thinking and convergent thinking, both of which lead to creative production (Cropley, 2006). Divergent thinking involves searching through various directions, and multiple solutions to a problem are generated; in convergent thinking, thought is directed to one correct or best solution (Guilford, 1956, 1959).

Despite the growing number of studies done on creativity, there is still much to be learned (Runco and Albert, 2010). Throughout the years, researchers have studied creativity from various perspectives, including how individuals’ beliefs influence creativity. The topic of beliefs about creativity has been approached from different angles such as how people view themselves (i.e., creative self-beliefs) and how people perceive the nature of creativity. In this paper, we focuses on creative self-efficacy which is one of the key self-beliefs, and beliefs about the malleable nature of creativity (i.e., creative mindsets) which have attracted more researchers recently.

Creative self-efficacy is the belief that one can produce creative outcomes (Tierney and Farmer, 2002). As in most fields, research on creative self-efficacy has been grounded in Bandura’s (1977) work on self-efficacy beliefs. Within this framework, self-efficacy beliefs determine how efficient people function through cognitive, motivational, affective, and decisional processes (Bandura, 1993, 2011). Self-beliefs of efficacy influence how much effort people put into a task, how persistent they are, and what task choices they prefer (Bandura, 1977; Zimmerman, 2000b; Schunk and DiBenedetto, 2016). When facing a challenge, people gauge their capacity to keep themselves motivated, focus on the task at hand, and manage negative thoughts and feelings (Bandura and Locke, 2003). Self-efficacy and performance mutually influence each other (Bandura, 1989; Williams and Williams, 2010). Past experiences shape people’s current beliefs and their current beliefs drive their future actions.

Previous research has revealed evidence of the association between creative self-efficacy and creativity as assessed by various measures. For instance, in organizational settings, Michael et al. (2011) found that employees’ creative self-efficacy was positively related to their self-reported innovative behaviors. Studies by Tierney and Farmer (2002, 2011) also demonstrated that employees with high levels of creative self-efficacy tended to be rated with high levels of creativity by their supervisors as well. In school contexts, Beghetto et al. (2011) investigated elementary school students’ self-efficacy in creativity and found more self-efficacious students were given higher ratings of creative expression by their teachers. Karwowski (2011) studied high school and gymnasia students’ creative self-efficacy. Using an unfinished, framed drawing task as a measure of divergent thinking, Karwowski also found a positive link between students’ self-efficacy and their performance of the task. Based on prior research, the connection between creative self-efficacy and creativity is quite promising.

Unlike creative self-efficacy, creative mindsets are not self-beliefs but rather implicit theories concerning the source and nature of creativity (Karwowski and Brzeski, 2017). The work of Dweck and her colleagues on malleability beliefs has guided research on creative mindsets (e.g., Dweck and Leggett, 1988; Mueller and Dweck, 1998; Hong et al., 1999). According to their research, it makes a difference whether people believe that a certain attribute is fixed or unchangeable (fixed beliefs) or that a certain attribute is developable through hard work (incremental beliefs). When engaging in a task, people with fixed beliefs attribute their success or failure to the presence or lack of ability; conversely, people with incremental beliefs ascribe the task outcome to effort (Hong et al., 1999; Haimovitz and Dweck, 2017). As such, holding incremental beliefs is linked to desirable behaviors such as persistence, adoption of adaptive goals, and resilience in the face of setbacks (Mueller and Dweck, 1998; Yeager and Dweck, 2012). Holding fixed beliefs, on the other hand, is related to maladaptive behaviors such as learned helplessness (Hong et al., 1999). Compared to fixed beliefs, therefore, incremental beliefs lead to achievement in the long term (Blackwell et al., 2007). Dweck (2006) has introduced the terms “growth mindsets” and “fixed mindsets.” People with incremental beliefs endorse a growth mindset, while people with fixed beliefs endorse a fixed mindset. In this paper, the term “creative mindsets” is used to refer to beliefs concerning the malleable nature of creativity.

The concept of creative mindsets is relatively new. As a result, the connections between creative mindsets and creativity have been explored less than creative self-efficacy has. O’Connor et al. (2013) conducted a series of studies to examine creative mindsets and creativity. Using their self-developed scale, they found that the creative growth mindset positively predicted interest in creative thinking, creative performance as assessed by the Unusual Uses Task (also known as the Alternative Uses Task), self-reported creativity (Study 1), and prior creative achievements across various domains (Study 2). Manipulation of creative mindsets (Study 3) also demonstrated that participants in the growth-mindset-induced group performed better in the Unusual Uses Task. This study provided evidence that creative mindsets affect creative performance. Karwowski (2014) developed a scale to measure creative mindsets and examined their relations to creative problem-solving as measured by insight problems. He found that the fixed mindset was related to inefficient problem-solving performance.

Besides using different instruments to measure creativity and creative mindsets, O’Connor et al. (2013) and Karwowski (2014) viewed two types of mindsets differently in terms of their constructs. The research done by O’Connor et al. (2013) was based on the premise that people endorse either fixed beliefs or incremental beliefs. That is, growth and fixed mindsets together form one construct. This view is in accordance with the research done by Dweck and her colleagues (e.g., Hong et al., 1999; Blackwell et al., 2007). However, Karwowski (2014) argued that people can hold two kinds of mindsets simultaneously, which means that the fixed mindset and the growth mindset should be conceived of as two correlated yet separate constructs. This view has been supported by correlational results of factor analyses conducted by Hass et al. (2016), who found a negative correlation between fixed mindsets and growth mindsets, but the correlation was too small for the two to be considered as one construct. Furthermore, they found a positive correlation between the creative growth mindset and self-efficacy, but not between the fixed mindset and self-efficacy. As such, they concluded that while the two mindsets are related, they are indeed two distinct constructs. Additionally, applying a bifactor modeling approach and a latent profile analysis, Karwowski et al. (2018b) demonstrated that people can hold both fixed and growth mindsets. In fact, their results showed that people could be classified as people as those with high growth and low fixed mindsets, those with low growth and high fixed mindsets, those with high growth and high fixed mindsets, and those with low fixed and malleable mindsets.

Overall, evidence from past research has established the associations between these two types of beliefs about creativity and creativity. Specifically, high creative self-efficacy and growth mindset, rather than fixed mindset, appear to be linked to desirable creative outcomes. However, some inconsistencies regarding how researchers have hypothesized the direction of the associations should be addressed, especially if studies have involved creativity tasks. For instance, Karwowski (2011) used a creativity task, specifically a divergent thinking task, to study the association between creative self-efficacy and creativity. In his study, the performance in the task was treated as a predictor of self-efficacy. The direction of the divergent thinking performance and self-efficacy found in this study is in alignment with Karwowski and Beghetto’s (2018) Creative Behavior as Agentic Action model, which proposes that the link between creative potentials and creative achievement is mediated and moderated by creative confidence and valuing creativity. According to this model, divergent and convergent thinking abilities are viewed as creative potentials and essentially these abilities influence self-efficacy. Creative mindsets were later included in the Elaborated Creative Behavior as Agentic Action model (Karwowski et al., 2016). According to this model, creative mindsets influence the relationships between creative potential, creative self-beliefs, and creative behavior. In this later model, divergent and convergent thinking are also perceived as creative potentials which are neither predictors of self-efficacy nor creative mindsets. Conversely, some studies on creativity’s relationship with creative mindsets examined performance in a divergent thinking task, such as the Alternative Uses Task (O’Connor et al., 2013) or a convergent thinking task (e.g., insight problems; Karwowski, 2014), as an outcome of creative mindsets. This indicates that performance in divergent or convergent thinking tasks can be used as both a predictor and an outcome of beliefs. This difference may simply depend on how researchers view the performance of the tasks. As a predictor, performance may serve as a reference for people to evaluate their abilities and form their beliefs. As an outcome, performance represents some form of creative behavior which is a result of how beliefs influence actions. The present research is based on the premise that beliefs influence creative performance and it aims to explore some psychological factors that could potentially explain this mechanism.

### Potential Mediators Between Beliefs and Creativity

#### Cognitive Processing Channels

The dual pathway to creativity model asserts that creativity can be achieved through two cognitive pathways, namely the flexibility pathway and the persistence pathway (De Dreu et al., 2008; Nijstad et al., 2010). In the flexibility pathway, creativity is obtained through cognitive flexibility: that is, flexibly switching from one perspective to another (Nijstad et al., 2010). In the persistence pathway, creativity is accessed through cognitive persistence: in other words, through sustained and focused task-directed cognitive effort (Nijstad et al., 2010). The use of cognitive flexibility manifests itself in divergent thinking when individuals engage in broad cognitive categories and frequently switch among categories during the thinking process. On the other hand, the use of the persistence pathway is apparent when individuals draw many ideas from a few categories. In divergent thinking tasks in which participants have to produce ideas to solve a problem, the number of categories used by participants functions as an indicator of cognitive flexibility, while within-category fluency or the number of ideas within a category is used to measure persistence (De Dreu et al., 2008; Roskes et al., 2012). According to this model, some states or traits facilitate cognitive flexibility, while others enhance cognitive persistence. For instance, when using a brainstorming task, De Dreu et al. (2008) found that cognitive flexibility (the number of categories used) mediated the effect of positive affective states on originality; while cognitive persistence (within-category fluency) mediated the effect of negative mood states on creative fluency. Although both cognitive pathways can lead to creativity, the persistence pathway is believed to be less effective compared to the flexibility pathway because it requires more cognitive resources (Roskes et al., 2012).

Self-efficacy beliefs (Schunk and Zimmerman, 1997; Bandura, 2011) and incremental beliefs (Dweck, 2000; Dweck and Master, 2008) promote self-regulation. This paper hypothesizes that these beliefs are associated with greater flexibility, and that these associations may be due to their links to self-regulation. On the one hand, self-regulation, which involves cyclically making adjustments as needed based on prior knowledge (Zimmerman, 2000a), is driven by task-switching ability, since this ability allows people to flexibly switch between means and goals when appropriate (Hofmann et al., 2012). If self-efficacy and incremental beliefs are linked to the effective self-regulatory process, and this process relies on cognitive flexibility, then these beliefs could be related to cognitive flexibility. On the other hand, self-efficacy and incremental beliefs influence adaptive reactions to a situation, such as sustaining positive affect in the face of setbacks, adopting approach-based orientations, and maintaining motivation (as discussed in the “*Self-Regulatory Responses*” section). Because these reactions are believed to be facilitators of flexible processing, the beliefs should be connected with cognitive flexibility in one way or another.

#### Self-Regulatory Responses

As previously mentioned, self-efficacy beliefs and malleability beliefs predict how people react to a situation. In this way, the beliefs predict achievement through the use of self-regulatory strategies. This paper hypothesizes that the same principle would apply to beliefs concerning creativity and creative achievement. More specifically, this paper hypothesizes that creative self-efficacy and creative mindsets affect creativity by triggering self-regulatory reactions that promote or demote creativity.

##### Affective states

The beneficial effects of beliefs on emotional regulation seem to be most apparent when individuals encounter challenging situations. Perceived self-efficacy has an impact on individuals emotionally (Linnenbrink and Pintrich, 2003). Past research has shown that people with a weak sense of self-efficacy are more vulnerable to negative emotional experiences such as childhood depression (Bandura et al., 1999), test anxiety (Komarraju and Nadler, 2013; Roick and Ringeisen, 2017), and job stress (Klassen and Chiu, 2010). With respect to creative self-efficacy, Rego et al. (2012) found that employees’ self-efficacy beliefs were positively correlated with positive affect, and that positive affect partially mediated the relationship between self-efficacy and creativity as rated by their supervisors.

With respect to malleability beliefs, research devoted to intelligence among students revealed that students who think intelligence is undevelopable are likely to experience negative feelings such as anxiety, anger, shame, hopelessness, and boredom (King et al., 2012). In the sports domain, Gardner et al. (2015) found that people with a stronger fixed mindset were more vulnerable to competition anxiety, whereas a stronger growth mindset was related to less anxiety. The unfavorable impacts of fixed beliefs could be explained by their association with less effective emotion regulation (Schroder et al., 2015). Given that creative mindsets have been built on the same foundation as other areas, their connections with affect should appear indifferent. That is a fixed mindset would be associated with negative affect and a growth mindset would be related to positive affect.

As previously mentioned, creativity can be achieved via flexibility and persistence pathways, with flexibility being the preferable pathway. Both positive affect and negative affect can lead to creativity as long as they are activating (De Dreu et al., 2008; Nijstad et al., 2010). Positive activating affect facilitates cognitive flexibility; on the other hand, negative activating affect increasing the use of cognitive persistence. Based on past research, it seems that when performing a creativity task, people with high creative self-efficacy and a growth mindset would experience lower negative affect and higher positive affect, which would lead to flexible thinking and creativity, while a fixed mindset would result in the opposite outcomes.

##### Approach/avoidance orientation

When engaging in a task, people with a strong sense of self-efficacy anticipate success, while those who perceive low self-efficacy visualize failure (Bandura, 1993). Inefficacious people are therefore apt to see task demands as threats to be avoided rather than challenges to be learned from (Chemers et al., 2001). Past research on achievement goals has provided some evidence on the impact of self-efficacy beliefs on approach/avoidance orientations. For instance, studies in educational settings have shown that students with high self-efficacy tend to adopt approach-based goals such as mastering a given task or demonstrating their competence (Pajares et al., 2000; Cury et al., 2006; Van Yperen, 2006; Komarraju and Nadler, 2013). Conversely, students with low self-efficacy are prone to engage in avoidance-based goals such as avoiding showing their incompetence (Pajares et al., 2000; Cury et al., 2006; Van Yperen, 2006). With respect to creative self-efficacy, research done by Beghetto (2006) and Puente-Díaz and Cavazos-Arroyo (2017) has revealed a similar trend in which people with high creative self-efficacy tend to engage in approach orientations.

Malleability beliefs influence what types of goals people adopt, but unlike self-efficacy, they seem to be unable to predict the engagement of approach/avoidance orientations. Research has indicated that people who hold a fixed mindset are likely to adopt both approach-based goals such as demonstrating their competence (Robins and Pals, 2002; Cury et al., 2006) and avoidance-based goals such as avoiding showing their incompetence (Cury et al., 2006). On the other hand, those that hold a growth mindset have a tendency to adopt approach-based goals such as learning or mastering a subject (Robins and Pals, 2002; Cury et al., 2006; Lou and Noels, 2016) and avoidance-based goals such as avoiding learning less than they could (Cury et al., 2006). In the case of creative mindsets, a recent study by Puente-Díaz and Cavazos-Arroyo (2017) revealed that the growth mindset and the fixed mindset were both positively related to approach-based goals. Evidently, mindsets predict what types of goals people prefer, but not the approach/avoidance orientation of the goals.

Because approach orientations are linked to higher cognitive flexibility, (Nijstad et al., 2010; Roskes et al., 2012), this paper hypothesizes that people with high creative self-efficacy will adopt an approach orientation, which will then enhance cognitive flexibility and subsequently creativity.

##### Task enjoyment

Task enjoyment/interest is an indicator of intrinsic motivation (Ryan, 1982; Davis et al., 1992; Ryan and Deci, 2000). In fact, the use of self-reported interest and enjoyment of the activity is a common approach to assessing intrinsic motivation (Ryan and Deci, 2000). Intrinsic motivation is when people are driven to engage in an activity because they find it interesting or enjoyable (Amabile and Pillemer, 2012). This type of motivation is involved in cognitive flexibility (Deci and Ryan, 2000), and is believed to be conducive to creativity (Amabile and Pillemer, 2012).

Perception of ability has been positively linked to motivation (Bandura, 1993, 2011). For example, early work by Bandura and Schunk (1981) found that students with higher mathematical self-efficacy were more intrinsically interested in arithmetic tasks. Zimmerman and Kitsantas (1997) also found that perceived self-efficacy in dart skills was positively correlated with interest in the game. Similar results have been found in sports literature. Hu et al. (2007) provided participants with fake feedback on their exercise tests to manipulate their self-efficacy in exercise. The results showed that people in the high-self-efficacy group enjoyed their physical activity more than their counterparts in the low-self-efficacy group.

As for studies concerning malleability beliefs, the same trend has been found in people with a growth mindset. For instance, in a study by Aronson et al. (2002), participants who were convinced that intelligence was improvable through hard work reported that they experienced greater enjoyment during academic processes. The impact of incremental beliefs on enjoyment even persists after setbacks. Mueller and Dweck (1998) demonstrated that praising students for their hard work (promoting growth mindsets) rather than their intelligence (promoting fixed mindsets) helped to sustain their task enjoyment even after facing failure. With respect to creativity research, O’Connor et al. (2013) also found a positive correlation between the creative growth mindset and self-reported interest in creative thinking.

Taking this all into consideration, this paper hypothesizes that self-perceived efficacy and creative mindsets will impact creativity via enjoyment of the task and the use of flexible processing.

##### Effort

Effort reflects how much people engage in an activity. Research literature emphasizes that exerting more effort is an adaptive behavioral outcome of self-efficacy beliefs (Bandura, 1977; Zimmerman, 2000b) and malleability beliefs (Dweck, 2000). Effort is a more controllable factor in comparison with ability. The extent of effort put forth depends on people’s own will, so if they are convinced that their accomplishments rely on their hard work, they tend to be more motivated to work harder (Schunk, 1983).

Research has suggested that people with high self-efficacy are likely to have a positive attitude toward effort. In the presence of challenges, self-efficacy predicts how long people persevere and how much energy they invest in a task (Zimmerman, 2000b; Pajares and Schunk, 2002; Bandura, 2011). After applying both questionnaire and diary methods to assess academic effort, Trautwein et al. (2009) reported a positive association between effort and self-competence beliefs. Similarly, Komarraju and Nadler (2013) found positive correlations among undergraduate students’ grade point average, self-efficacy, and effort regulation (working hard and persisting when necessary). Their mediation analysis also demonstrated that effort regulation partially mediated the link between self-efficacy and academic achievement.

With respect to malleability beliefs, research suggests that by valuing hard work, people with a growth mindset expend more effort on tasks. For instance, Mueller and Dweck (1998) demonstrated that when students were praised for their ability, they tended to view their performance as the outcome of their ability instead of their effort, and so when given a choice, they were less willing to spend more time on the activity. Hong et al. (1999) provided participants with a false negative result of a task that allegedly tested their intelligence. Manipulating participants’ fixed and growth mindsets, they found that those in the growth-mindset group were prone to ascribe the outcome to effort, and they were apt to express willingness to take remedial action.

To a certain extent, creativity requires conscious effort (Cropley, 2006). Conscious effort is involved with creative production in the preparation process (Busse and Mansfield, 1980; Cropley, 2006) in the way that it enables and provides direction to unconscious creative processing (Busse and Mansfield, 1980). As such, effort may mediate the links between the beliefs and creative performance.

### The Present Research

The two present studies were intended to explore factors that could explain how creative self-efficacy and creative mindsets impact creativity. Creative self-efficacy and mindsets, more specifically the growth mindset, have been found to be correlated with each other (Karwowski, 2014; Hass et al., 2016), yet the causal aspect of the relationship is not clear. For instance, highly self-efficacious people might experience more success, thus believing that their ability can be improved. Holding a growth mindset might motivate people to work harder, help them gain more achievements, and consequently boost their self-efficacy. Given that the direction of self-efficacy and creative mindsets was not our focus, we therefore treated both of them equally as predictors of creative performance. Furthermore, the relationships between beliefs and creative performance was not our main interest; instead, our main concern was to explore factors that could link them.

As previously mentioned, creativity can be achieved by adopting flexible cognitive processing and persistent processing. Because these two processing styles manifest themselves in divergent thinking, we applied a divergent thinking task in Study 1. To test if creative beliefs may impact creativity through either or both cognitive flexibility and persistence, we treated these two traits as mediators. Additionally, self-efficacy and ability mindsets determine how people regulate themselves when facing a challenge (through self-regulatory responses). Previously, we proposed some self-regulatory responses that affect creativity in a positive way. There is also some evidence suggesting that adopting certain self-regulatory responses may lead to different types of cognitive processing. Accordingly, we treated these self-regulatory reactions as another set of mediators, and tested whether they could connect beliefs with creative performance directly and/or connect them with creative performance indirectly, through different types of cognitive channels. In Study 2, we adopted a convergent thinking task instead, to examine if beliefs would impact this task in the same way. However, due to the task type, cognitive flexibility and persistence scores could not be computed, and are thus not included in the examination.

## Study 1

### Participants

The participants were upper secondary school students recruited from schools in Thailand. Originally, 276 students participated in this study. Nine cases were excluded due to missing data. Fourteen cases were excluded due to unengaged responses. Five cases were excluded due to misunderstanding the instruction of the creativity task. The final sample thus consisted of 248 students with a mean age of 16.97 (*SD* = 1.07). Of this sample, 157 were female, and all were native Thai speakers. The research is approved by the University Committee on Human Research Protection (UCHRP) of East China Normal University. In addition, permission from the schools’ principals and consent forms from participants and their parents/guardians were obtained prior to data collection.

### Measures

The questionnaires employed in this study were originally written in English. In order to administer the questionnaires to this particular sample, the back-translation technique recommended by Brislin (1986) was applied. First, one of the authors translated the questionnaire items from English to Thai, and a professional English–Thai translator blindly to the original content translated them back to English. The back-translated and original versions were later compared to determine whether or not the concepts were different. Problematic items were adjusted via discussion between the two translators. To ensure that the translated questionnaires were comprehensible to the target sample, four upper secondary school students were asked to complete them and provide feedback. Again, problematic items were adjusted via discussion between the two translators.

#### Beliefs Concerning Creativity

##### Creative self-efficacy

Creative self-efficacy was measured using six items of creative self-efficacy subscale from the Short Scale of Creative Self Scale (Karwowski et al., 2018a). Participants responded on a 5-point Likert scale with 1 being “strongly disagree” and 5 being “strongly agree.” One sample item is, “I am good at proposing original solutions to problems.”

##### Creative mindsets

Creative mindsets were measured using a 5-point Likert (1 being “strongly disagree” and 5 being “strongly agree”) developed by Karwowski (2014). The scale consists of a 5-item fixed mindset subscale (“You either are creative, or you are not—even trying very hard you cannot change much”) and a 5-item growth mindset subscale (“Everyone can create something great at some point if he or she is given appropriate conditions”).

#### Creativity

The Alternative Uses Task was used to assess creativity associated with divergent thinking. In this task, participants were given 10 min to come up with creative uses for a brick. Three scores (originality, flexibility, and persistence) were computed from this task. *The originality score* was the number of responses that were provided by less than 5% of all participants. A high score indicated high creativity. *The flexibility score* was the number of categories used. A high score reflected high cognitive flexibility (e.g., De Dreu et al., 2008; Roskes et al., 2012). *The persistence score* was the number of responses divided by the flexibility score. A high score represented high cognitive persistence (e.g., De Dreu et al., 2008). As such, only the originality score was used to represent creative performance, while the flexibility score and the persistence score were used to indicate cognitive processing tendencies.

#### Self-Regulatory Responses

##### Affective states

Affective states during the task were assessed using the Positive and Negative Affect Schedule (PANAS; Watson et al., 1988). The scale consists of 10 items of mood descriptors evaluating positive affect (PA) and 10 items of mood descriptors evaluating negative affect (NA). These two dimensions were later renamed positive activation and negative activation due to the activating nature of the mood descriptors used in the scale (Watson et al., 1999). Participants had to indicate on a scale of 1 to 7 (1 being “not at all” and 7 being “extremely”) to what extent they felt a specific mood during the creativity task. These scales were to be completed after the creativity task.

##### Approach/avoidance orientation

A force-choice approach was employed to assess approach orientation versus avoidance orientation. This approach was used successfully in prior studies to measure approach/avoidance achievement goals (e.g., Mueller and Dweck, 1998; Van Yperen and Renkema, 2008). Participants in this study were forced to choose one of the two statements that was the most accurate for them. The two statements were “During the task, I focused on performing well,” representing the approach orientation, and “During the task, I focused on not performing poorly,” representing the avoidance orientation. For analysis purposes, the avoidance orientation and the approach orientation were coded as 0 and 1, respectively.

##### Task enjoyment

Seven items from the Interest/Enjoyment Subscale from the Intrinsic Motivation Inventory (Ryan, 1982) were used as a measure of task enjoyment. A sample item of this subscale is “I enjoyed doing the task very much.” Participants had to respond on a scale of 1 to 7 with 1 being “not true at all” and 7 being “extremely true.”

##### Effort

Effort exerted during the creativity task was measured using the Effort/Importance Subscale from the Intrinsic Motivation Inventory (Ryan, 1982). Out of 5 items, 1 item of this subscale measures importance. For this study, this item was excluded and the remaining four items were used to measure effort. A sample item is “I put a lot of effort into the task.” Participants had to respond on a scale of 1–7 with 1 being “not true at all” and 7 being “extremely true.”

#### Other Variables

##### Valence and arousal

The valence and arousal scales from Lang’s (1980) Self-Assessment-Manikin were used to measure valence and arousal dimensions of affective states. Participants were asked to complete these scales before engaging in the creativity task to measure their pre-existing affective states.

##### Age

The participants’ ages were asked as one of the demographic questions.

### Procedure

The questionnaires and the creativity task were paper-based and administered in a classroom to groups of 20 to 30 participants at a time. Participants were asked to complete the demographic questions first, followed by the scales measuring pre-task affective states, creative mindsets, and creative self-efficacy. Participants then worked on the creativity task. Lastly, approach/avoidance orientations, affective states, task enjoyment, and effort were measured.

### Results and Discussion

This study employed the structural equation modeling (SEM) technique for statistical analyses using Mplus version 7.4. SEM is a multivariate method that allows researchers to test a series of dependence relationships at the same time (Hair et al., 2010). Given that this study dealt with multiple variables, this method was suitable for the present data.

In this study’s SEM models, all three creativity scores (originality, flexibility, and persistence), age, and valence and arousal were treated as continuous variables. Of all these variables, kurtosis values of the originality score and the persistence score were outside the acceptable range of ±2 (Lomax and Hahs-Vaughn, 2012). Log-transformation was therefore performed for the persistence score, and because the originality score contained zero values, square-root transformation was performed instead. Kurtosis values of these two variables fell within the acceptable range after the transformation. Approach/avoidance orientation was a binary variable. While indicators that are Likert-scale responses with five categories or more are generally treated as continuous variables, the histograms of our scale responses revealed some floor and ceiling effects. Treating indicators with asymmetrical distribution as continuous is not appropriate (Kline, 2016); therefore, responses in the scales of creative self-efficacy, creative mindsets, task enjoyment, and effort were defined as ordered-categorical variables. Analyses were employed using mean-and-variance-adjusted weighted least squares estimation (WLSMV) to account for non-continuous variables. With this estimation method, the regression coefficients produced are linear regression coefficients when dependent variables are continuous or continuous latent; the regression coefficients are probit regression coefficients when dependent variables are binary or ordered categorical (Muthén and Muthén, 1998–2012). Fit indices and criteria used were χ^2^*/df* for the parsimonious fit with value < 3 (Marsh and Hocevar, 1985; Hair et al., 2010), comparative fit index (*CFI*) for the incremental fit with values > 0.90 (Bentler, 1990; Hair et al., 2010), and root mean square error of approximation (*RMSEA*) for the absolute fit with value < 0.08 (Browne and Cudeck, 1993).

#### Test of the Measurement Model

Before proceeding with SEM, a confirmatory factor analysis (CFA) was performed to validate the measurement model of seven latent constructs: creative self-efficacy, fixed mindset, growth mindset, negative affect, positive affect, task enjoyment, and effort. Items loaded on their perspective factors smaller than 0.35 were dropped to improve unidimensionality. Accordingly, 1 item from the fixed mindset scale, 4 items from the positive affect scale, and 1 item from the effort scale were removed. The final model yielded an acceptable fit [χ^2^(758) = 1490.29, *p* < 0.001, χ^2^*/df* = 1.97, *CFI* = 0.92, and *RMSEA* = 0.06]. The reliability coefficients of these scales, along with descriptive statistics and correlations among the latent and observed variables, are presented in **Table 1**.

**Table 1:** Descriptive statistics, reliability coefficients, and correlations for Study 1.

	CSE^a^	CGM^a^	CFM^a^	PA^a^	NA^a^	TE^a^	Ef^a^	Ap/Av	Or	Pe	Fl
CGM	0.32***										
CFM	-0.04	-0.52***									
PA	0.42***	0.37***	-0.24**								
NA	-0.06	-0.18*	0.27***	-0.05							
TE	0.29***	0.19**	-0.09	0.61***	-0.2***						
Ef	0.37***	0.29***	-0.16*	0.48***	-0.21***	0.56***					
Ap/Av	0.17	0.17	0.05	0.08	-0.02	0.10	0.12				
Or	0.24**	0.11	-0.14*	0.25***	-0.02	0.17**	0.16*	-0.02			
Pe	0.00	-0.02	0.08	0.05	0.00	0.07	-0.04	0.01	0.14*		
Fl	0.28***	0.16*	-0.2**	0.34***	-0.08	0.22**	0.21**	0.04	0.62***	-0.34***	
*CR*	0.80	0.76	0.68	0.81	0.92	0.93	0.86				
α	0.75	0.67	0.63	0.78	0.87	0.90	0.80				
*M*	3.23	4.35	2.41	4.60	2.07	4.81	5.31		3.37	2.87	4.56
*SD*	0.49	0.48	0.65	0.89	0.90	0.97	1.12		2.85	1.39	1.72
Frequency (%)								Ap : 167 (67.34)			


**Correlation coefficients were estimated based on Model 3. ^∗^p < 0.05, ^∗∗^p < 0.01, ^∗∗∗^p < 0.001; CSE, creative self-efficacy; CGM, creative growth mindset; CFM, creative fixed mindset; PA, positive affect; NA, negative affect; TE, task enjoyment; Ef, effort; Ap, approach orientation; Av, avoidance orientation; Or, originality score; Pe, persistence score; Fl, flexibility score; CR, composite reliability; α, Cronbach’s alpha; M, means; SD, standard deviations. ^*a*^The means of these scales were calculated as though their indicators were continuous variables. However, these indicators were actually treated as ordered-categorical variables in the SEM models.**

Next, the relationships among the variables were tested using a series of SEM models. To control the effects of age and affective states prior to engaging in the creativity task on dependent variables, age and valence and arousal were entered into all SEM models as covariates (i.e., all endogenous variables were regressed on these variables).

#### Effects of Beliefs on Creativity

The effects of creative self-efficacy and the two kinds of creative mindsets on creativity were first investigated. Model 1, comprising of creative self-efficacy and the two types of creative mindsets as predictor variables, and the originality score as the only outcome variable, demonstrated an adequate fit [χ^2^(135) = 242.54, *p* < 0.001, χ^2^*/df* = 1.80, *CFI* = 0.90, and *RMSEA* = 0.06]. No trimming was performed. To account for possible multicollinearity among independent variables, variance inflation factors (VIFs) were computed. A variable may constitute a problem, if the VIF is greater than 10 (Kline, 2016). The results suggested that multicollinearity was not an issue among the predictors (VIFs range: 1.15–1.58). Based on this model, creative self-efficacy was found to positively predict originality (β = 0.24, *p* = 0.001), indicating that the more people believe they have capacities to be creative, the more likely they are to produce creative ideas. A study by Karwowski (2011) also found this positive relationship between creative self-efficacy and divergent thinking. Effects of creative growth and fixed mindsets on originality were not observed (β = -0.06, *p* = 0.502 and β = -0.17, *p* = 0.055, respectively). Research literature emphasizes the role of mindsets when facing setbacks (Dweck, 2006). As such, their role may be limited when it comes to relatively easy tasks (Karwowski et al., 2016). Given that the Alternative Uses Task is not a very challenging task, it might not allow the effects of mindsets to manifest themselves.

#### Effects of Beliefs on Creativity via Cognitive Processing Channels

Creativity can be achieved by being cognitively flexible or/and being cognitively persistent (Nijstad et al., 2010). The flexibility score and the persistence score were therefore inserted into the model as mediators (Model 2). In this model, the scores of originality, flexibility, and persistence were regressed on creative self-efficacy and the two creative mindsets. The originality score was also regressed on the flexibility and persistence scores. Following Preacher and Hayes’s (2008) recommendation, residuals of the mediators were covaried. The model yielded an acceptable fit [χ^2^(159) = 265.89, *p* < 0.001, χ^2^*/df* = 1.67, *CFI* = 0.91, and *RMSEA* = 0.05], and no trimming was performed. Examination of VIFs suggested multicollinearity among the predictors and mediators was not a concern (VIFs range: 1.17–1.58). Indirect effects were tested using the *model indirect* command in Mplus. With this command, indirect effects are defined as products of regression coefficients (Muthén and Muthén, 1998–2012).

Results showed that persistence and flexibility positively predicted originality (β = 0.40, *p* < 0.001 and β = 0.74, *p* < 0.001, respectively). Results also showed that creative self-efficacy positively predicted flexibility (β = 0.29, *p* < 0.001), but unlike Model 1 no longer had a significant effect on originality (β = 0.04, *p* = 0.435). A significant indirect effect of creative self-efficacy on originality via flexibility was detected (β = 0.21, *p* = 0.001). These results demonstrate that the effect of self-efficacy on originality was fully mediated through flexibility, indicating that people produce creative ideas by engaging in flexible processing when they are self-efficacious in their creativity.

**FIGURE 1:**
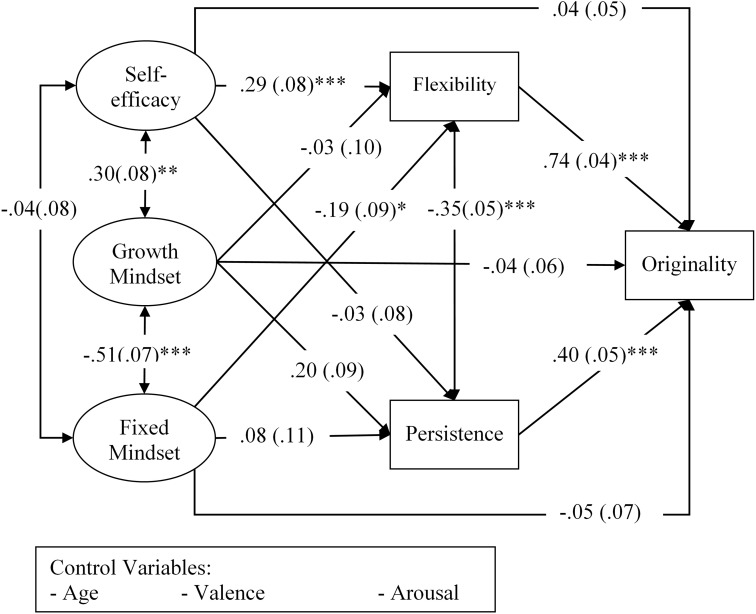
The relationships between creativity as indicated by the originality score and, creative self-efficacy and creative mindsets via cognitive processing channels while controlling the effects of age, valence and arousal (Model 2). The values represent standardized path coefficients with standard errors in parentheses. ^∗^*p* < 0.05, ^∗∗^*p* < 0.01, ^∗∗∗^*p* < 0.001.

Additionally, flexibility was also predicted by the fixed mindset, but in a negative direction (β = -0.19, *p* = 0.026). An indirect effect of fixed mindset on originality via flexibility also appeared significant (β = -0.14, *p* = 0.03), indicating that people who hold a low level of fixed mindset tend to be more cognitively flexible, and this in turn leads to more original ideas. Results from the growth mindset failed to emerge. **Figure 1** illustrates Model 2 with path coefficients.

#### Effects of Beliefs on Creativity via Self-Regulatory Responses

**FIGURE 2:**
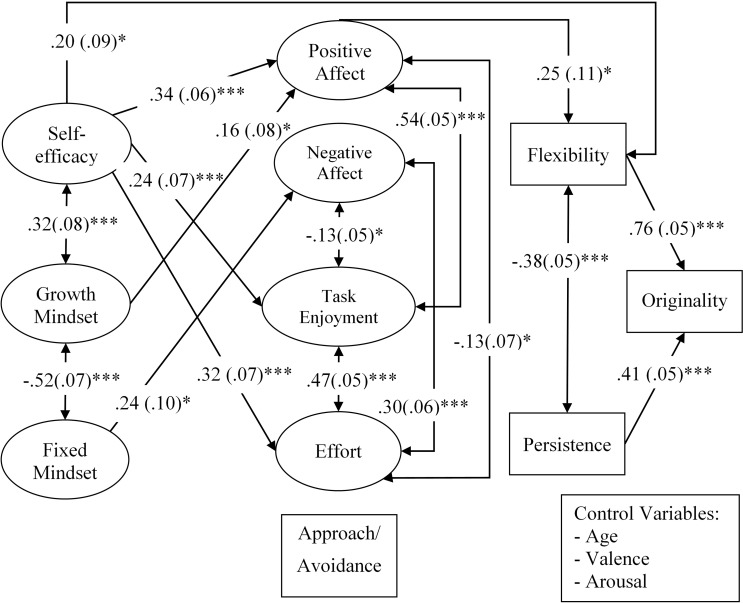
The relationships between creativity as indicated by the originality score and, creative self-efficacy and creative mindsets via cognitive processing channels and self-regulatory responses while controlling the effects of age, valence and arousal (Model 3). For the sake of clarity, only significant direct paths are displayed. The values represent standardized path coefficients with standard errors in parentheses. ^∗^*p* < 0.05, ^∗∗∗^*p* < 0.001.

To test whether or not any proposed self-regulatory responses (i.e., positive affect, negative affect, approach/avoidance orientation, task enjoyment, and effort) could explain the connections between the beliefs and creativity, these variables were added into the model (Model 3, as illustrated in **Figure 2**). For this model, all self-regulatory responses and the creativity scores (i.e., originality, flexibility, and persistence) were regressed on creative self-efficacy and the two mindsets. The creativity scores were regressed on all self-regulatory responses. The originality score was also regressed on the flexibility score and the persistence score. Again, residuals of parallel but not serial mediators were covaried. The model fit indices were satisfactory [χ^2^(996) = 1763.69, *p* < 0.001, χ^2^*/df* = 1.77, *CFI* = 0.91, and *RMSEA* = 0.06]. No trimming was performed and multicollinearity among the predictors and mediators was not a concern (VIFs range: 1.09–2.20).

After examining path coefficients, the results demonstrated that creative self-efficacy positively predicted positive affect (β = 0.34, *p* < 0.001), task enjoyment (β = 0.24, *p* < 0.001), and effort (β = 0.32, *p* < 0.001). As in Model 2, the direct effect of creative self-efficacy on flexibility remained significant (β = 0.20, *p* = 0.03). With regard to creative mindsets, the fixed mindset appeared to be a positive predictor of negative affect (β = 0.24, *p* = 0.015). Inconsistent with Model 2, the direct effect of the fixed mindset on flexibility became insignificant (β = -0.15, *p* = 0.101). In addition, the growth mindset appeared to positively predict positive affect (β = 0.16, *p* = 0.04). The results were in line with prior studies demonstrating the beneficial effects of high self-efficacy on affect (e.g., Rego et al., 2012), task enjoyment (e.g., Hu et al., 2007), and effort (e.g., Trautwein et al., 2009), as well as the favorable effect of growth mindset and the adverse effect of fixed mindset on affect (e.g., King et al., 2012). With respect to the direct relationships of the proposed self-regulation related responses and creativity, only the positive relationship between positive affect and flexibility was observed (β = 0.25, *p* = 0.016). Additionally, persistence and flexibility remained positively related to originality (β = 0.41, *p* < 0.001 and β = 0.76, *p* < 0.001, respectively). All path coefficients of this model are displayed in **Table 2**.

**Table 2:** Direct effects of Model 3.

	Effects of CSE		Effects of CGM		Effects of CFM	
			
	β *(SE)*	*p*-value	β *(SE)*	*p*-value	β *(SE)*	*p*-value
On PA	0.34 (0.06)	<0.001	0.16 (0.08)	0.040	-0.16 (0.09)	0.069
On NA	-0.02 (0.07)	0.735	-0.05 (0.11)	0.649	0.24 (0.10)	0.015
On TE	0.24 (0.07)	<0.001	0.08 (0.09)	0.385	-0.05 (0.09)	0.560
On Ef	0.32 (0.07)	<0.001	0.12 (0.10)	0.221	-0.09 (0.09)	0.312
On Ap/Av	0.09 (0.10)	0.370	0.22 (0.13)	0.097	0.16 (0.13)	0.212
On Or	0.05 (0.06)	0.362	-0.02 (0.07)	0.757	-0.06 (0.07)	0.355
On Pe	-0.02 (0.09)	0.795	0.03 (0.11)	0.807	0.08 (0.11)	0.452
On Fl	0.20 (0.09)	0.030	-0.08 (0.10)	0.432	-0.15 (0.09)	0.101

	**Effects of PA**		**Effects of NA**		**Effects of TE**	
			
	**β** ***(SE)***	***p*-value**	**β *(SE)***	***p*-value**	**β *(SE)***	***p*-value**

On Or	-0.06 (0.10)	0.510	0.07 (0.05)	0.156	-0.03 (0.08)	0.688
On Pe	0.04 (0.12)	0.760	-0.02 (0.07)	0.782	0.10 (0.12)	0.402
On Fl	0.25 (0.11)	0.016	-0.02 (0.06)	0.699	0.01 (0.10)	0.933

	**Effects of Ef**		**Effects of Ap/Av**			
		
	**β *(SE)***	***p*-value**	**β *(SE)***	***p*-value**		

On Or	0.05 (0.06)	0.430	-0.06 (0.06)	0.286		
On Pe	-0.12 (0.09)	0.192	-0.01 (0.09)	0.915		
On Fl	0.01 (0.09)	0.930	0.02 (0.09)	0.827		

	**Effects of Pe**		**Effects of Fl**			
		
	*** β (SE)***	***p*-value**	**β *(SE)***	***p*-value**		

On Or	0.41 (0.05)	<0.001	0.76 (0.05)	<0.001		


*CSE, creative self-efficacy; CGM, creative growth mindset; CFM, creative fixed mindset; PA, positive affect; NA, negative affect; TE, task enjoyment; Ef, effort; Ap, approach orientation; Av, avoidance orientation; Or, originality score; Pe, persistence score; Fl, flexibility score.*

Again, the *model indirect* command in Mplus was employed to test indirect effects. As in Model 2, creative self-efficacy predicted originality via flexibility (β = 0.15, *p* = 0.034). The results also revealed that creative self-efficacy positively predicted flexibility via positive affect (β = 0.09, *p* = 0.023), and positive affect positively predicted originality via flexibility (β = 0.19, *p* = 0.02). The indirect path from creative self-efficacy to originality via positive affect and flexibility also appeared to be statistically significant (β = 0.07, *p* = 0.028). No other indirect effects were observed.

In summary, the relationship between creative self-efficacy and creativity as indicated by the originality score can be explained by flexibility and positive affect. More precisely, creative self-efficacy facilitates flexible thinking, which in turn enhances creativity. Additionally, creative self-efficacy also promotes positive affect, which partially increases cognitive flexibility. This is in alignment with the notion of the dual pathway to creativity model in which creativity can be achieved effectively through flexibility, and flexibility can be driven by positive affect (Nijstad et al., 2010). However, the effect of negative affect on creativity via persistence was not detected. This could be explained by the work of Roskes et al. (2012) suggesting that the persistence pathway costs more cognitive resources and people tend to exert these resources only when necessary. It is possible that participants in this study did not see the necessity of performing the task well. Therefore, their negative affect did not lead to creativity.

## Study 2

### Participants

Participants were upper secondary school students recruited from schools in Thailand. Initially, 239 students participated in this study. Of this number, 12 cases were excluded due to missing data and 13 cases were excluded due to unengaged responses. The final sample consisted of 214 students with a mean age of 17.05 (*SD* = 0.91). Among this sample, 116 students were female, and all were native Thai speakers. The research is approved by the University Committee on Human Research Protection (UCHRP) of East China Normal University. In addition, permission from the schools’ principals and consent forms from participants and their parents/guardians were obtained prior to data collection.

### Measures

All measures used in this study were the same as those used in Study 1, except for the creativity task. In this study, the Insight Problems Task was used to measure creativity associated with convergent thinking. Participants were presented with 10 insight problems. They were given 10 min to solve as many problems as possible. The number of correct answers was used as the indicator of creative problem-solving. Insight problems used in this study were adapted from Dow and Mayer (2004). A sample problem is “A woman’s earring fell into a cup that was filled with coffee, but her earring did not get wet. How could this be?”

### Procedure

The procedure was the same as that followed in Study 1.

### Results and Discussion

As in Study 1, an SEM analysis using Mplus version 7.4 was employed. Mean-and-variance-adjusted weighted least squares estimation was once again used to handle categorical and continuous data. The problem-solving score, age, and valence and arousal were treated as continuous variables. Approach/avoidance orientation was treated as a binary categorical variable, and responses of the other Likert scales were treated as ordered-categorical variables. Model fit indices and criteria were the same as those used in Study 1.

#### Test of the Measurement Model

Before proceeding with SEM, a CFA was conducted to validate the measurement model of seven latent constructs: creative self-efficacy, fixed mindset, growth mindset, positive affect, negative affect, task enjoyment, and effort. As in Study 1, items with factor loadings smaller than 0.35 were excluded to improve unidimensionality. As a result, 1 item from the fixed mindset scale, 1 item from the effort scale, 1 item from the negative affect scale, and 5 items from the positive affect scale were omitted. The final model showed an acceptable fit [χ^2^(681) = 1236.22, *p* < 0.001, χ^2^*/df* = 1.82, *CFI* = 0.95, and *RMSEA* = 0.06]. The scale reliability coefficients are presented in **Table 3** along with descriptive statistics and correlations among the latent and observed variables.

**Table 3:** Descriptive statistics, reliability coefficients, and correlations for Study 2.

	CSE^a^	CGM^a^	CFM^a^	PA^a^	NA^a^	TE^a^r	Ef^a^	Ap/Av	PS
CGM	0.55***								
CFM	-0.12	-0.34***							
PA	0.29***	0.59***	-0.25***						
NA	-0.24***	-0.28***	0.24**	-0.33***					
TE	0.25***	0.60***	-0.38***	0.79***	-0.42***				
Ef	0.45***	0.68***	-0.40***	0.74***	-0.25***	0.69***			
Ap/Av	0.25**	0.21*	-0.12	0.17	-0.06	0.25**	0.20*		
PS	0.18*	0.33***	-0.40***	0.25***	-0.16**	0.32***	0.31***	0.13	
*CR*	0.84	0.77	0.65	0.87	0.92	0.93	0.82		
α	0.80	0.66	0.62	0.84	0.88	0.90	0.75		
*M*	3.33	4.27	2.50	4.85	2.31	5.03	5.25	0.73	2.76
*SD*	0.52	0.50	0.66	0.99	1.00	1.08	1.12	0.44	1.87
Frequency (%)								Ap : 157 (73.36)	


*Correlation coefficients were estimated based on Model 5. ^∗^p < 0.05, ^∗∗^p < 0.01, ^∗∗∗^p < 0.001; CSE, creative self-efficacy; CGM, creative growth mindset; CFM, creative fixed mindset; PA, positive affect; NA, negative affect; TE, task enjoyment; Ef, effort; Ap, approach orientation; Av, avoidance orientation; PS, problem-solving score; CR, composite reliability; α, Cronbach’s alpha; M, means; SD, standard deviations. ^*a*^The means of these scales were calculated as though their indicators were continuous variables. However, these indicators were actually treated as ordered-categorical variables in the SEM models.*

Again, in SEM age, and valence and arousal were included in all models as covariates.

#### Effects of Beliefs on Creativity

To examine the associations between the beliefs and creativity, creative self-efficacy, fixed mindset, and growth mindset were added into the model as predictors, and the problem-solving score was entered into the model as the outcome variable (Model 4). The model demonstrated an acceptable fit [χ^2^(135) = 196.31, *p* < 0.001, χ^2^*/df* = 1.45, *CFI* = 0.95, and *RMSEA* = 0.05]. No trimming was performed. The VIF of each predictor was between 1.22 and 2.01, suggesting multicollinearity was not a problem.

The results demonstrated that the growth mindset positively predicted problem-solving performance (β = 0.26, *p* = 0.01). Conversely, the fixed mindset negatively predicted problem-solving (β = -0.31, *p* < 0.001). This result is in line with Karwowski’s (2014) finding, demonstrating a negative association between the fixed mindset and problem-solving. A significant link between creative self-efficacy and problem-solving did not emerge (β = 0.00, *p* = 0.98).

#### Effects of Beliefs on Creativity via Self-Regulatory Responses

**FIGURE 3:**
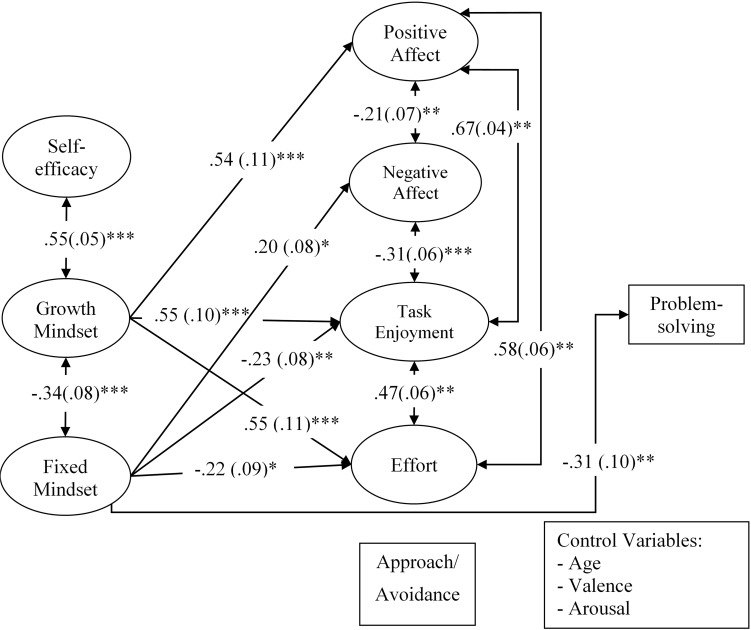
The relationships between creativity as indicated by the problem-solving score and, creative self-efficacy and creative mindsets via self-regulatory responses while controlling the effects of age, valence and arousal (Model 5). For the sake of clarity, only significant direct paths are displayed. The values represent standardized path coefficients with standard errors in parentheses. ^∗^*p* < 0.05, ^∗∗^*p* < 0.01, ^∗∗∗^*p* < 0.001.

All proposed self-regulatory responses (negative affect, positive affect, approach/avoidance orientation, task enjoyment, and effort) were introduced into the model (Model 5, as illustrated in **Figure 3**) to test whether or not they could explain how the beliefs are connected to creative problem-solving. For this model, all self-regulatory responses and the problem-solving score were regressed on creative self-efficacy and the two mindsets. The problem-solving score was also regressed on self-regulatory responses. All self-regulatory responses were entered into the model as parallel mediators and covaried. The model fit indices were acceptable [χ^2^(841) = 1403.54, *p* < 0.001, χ^2^*/df* = 1.67, *CFI* = 0.94, and *RMSEA* = 0.06]. No trimming was performed. Multicollinearity was not an issue among the predictors and mediators (VIFs range: 1.20–3.55).

Examining direct paths revealed that the fixed mindset negatively predicted task enjoyment (β = -0.23, *p* = 0.005) and effort (β = -0.22, *p* = 0.021), but positively predicted negative affect (β = 0.20, *p* = 0.013). Consistent with Model 4, the fixed mindset negatively predicted problem-solving (β = -0.31, *p* < 0.001). The growth mindset positively predicted task enjoyment (β = 0.55, *p* < 0.001), effort (β = 0.55, *p* < 0.001), and positive affect (β = 0.54, *p* < 0.001). Inconsistent with Model 4, the positive association between the growth mindset and the problem-solving score became insignificant (β = 0.17, *p* = 0.23). No significant effects from creative self-efficacy emerged. Path coefficients are presented in **Table 4**. These results were similar to those in Study 1 in terms of the adaptive effect of growth mindset and the maladaptive effect of fixed mindset on affect.

**Table 4:** Direct effects of Model 5.

	Effects of CSE		Effects of CGM		Effects of CFM	
			
	β *(SE)*	*p*-value	β *(SE)*	*p*-value	β *(SE)*	*p*-value
On PA	-0.03 (0.09)	0.728	0.54 (0.11)	<0.001	-0.10 (0.08)	0.207
On NA	-0.14 (0.09)	0.101	-0.11 (0.13)	0.373	0.20 (0.08)	0.013
On TE	-0.08 (0.07)	0.237	0.55 (0.10)	<0.001	-0.23 (0.08)	0.005
On Ef	0.13 (0.08)	0.131	0.55 (0.11)	<0.001	-0.22 (0.09)	0.021
On Ap/Av	0.20 (0.14)	0.145	0.04 (0.18)	0.824	-0.08 (0.13)	0.522
On PS	0.03 (0.11)	0.773	0.17 (0.14)	0.230	-0.31 (0.10)	0.001

	**Effects of PA**		**Effects of NA**		**Effects of TE**	
			
	**β *(SE)***	***p*-value**	**β *(SE)***	***p*-value**	**β *(SE)***	***p*-value**

On PS	-0.02 (0.15)	0.876	0.01 (0.06)	0.876	0.11 (0.14)	0.414

	**Effects of Ef**		**Effects of Ap/Av**			
		
	**β *(SE)***	***p*-value**	**β *(SE)***	***p*-value**		

On PS	0.01 (0.18)	0.972	0.02 (0.11)	0.849		


**CSE, creative self-efficacy; CGM, creative growth mindset; CFM, creative fixed mindset; PA, positive affect; NA, negative affect; TE, task enjoyment; Ef, effort; Ap, approach orientation; Av, avoidance orientation; PS, problem-solving score.**

Additionally, when testing for indirect effects using the *model indirect* command in Mplus, no significant results were detected indicating that the proposed self-regulatory responses cannot explain the connection between the beliefs and creative problem-solving.

Taken together, results suggest that creative growth and fixed mindsets may trigger some self-regulatory responses (i.e., affect, task enjoyment, and effort), albeit in the opposite direction. These responses, however, cannot account for the effectiveness of problem-solving.

## General Discussion

This research was intended to explore potential factors that could explain the associations between beliefs about creativity (i.e., creative self-efficacy and creative mindsets) and creative performance. Based on prior studies concerning self-efficacy and malleability beliefs, several related factors were proposed and tested for their mediating roles in the relationships between creative beliefs and creative production.

Study 1 investigated creativity associated with divergent thinking using the Alternative Uses Task as a measure. Results from SEM models demonstrated that creative self-efficacy positively predicted positive affect, task enjoyment, and effort. The growth mindset positively predicted positive affect. Conversely, the fixed mindset positively predicted negative affect. These results suggest that when engaging in a creativity task, people who feel more self-efficacious are likely to experience positive affect, enjoy the task more, and expend more effort. When people believe creativity can be improved, they, too, experience positive affect; however, when people see creativity as a fixed, unchangeable ability, they experience more negative affect. Overall, these results converge with past work outside of the topic of creativity that suggests that self-efficacy beliefs (Bandura, 1977; Pajares, 2008) and growth mindsets rather than fixed mindsets (Dweck, 2000; Molden and Dweck, 2006; Dweck and Master, 2008) are linked to beneficial self-regulatory outcomes.

Assessing the indirect effects of the beliefs on creativity revealed the mediation effect of flexibility on the relationship between creative self-efficacy and creativity, suggesting that participants with higher self-efficacy were more capable of producing creative ideas by being more cognitively flexible as reflected by the number of categories used during the task. This could be because self-efficacy is closely related to self-regulation. When people have a strong sense of self-efficacy, they self-monitor and adapt strategies as needed (Schunk and Zimmerman, 1997; Zimmerman, 2000a). As such, it is possible that, during the task, participants who were more self-efficacious were more successful in shifting from the old means that did not work to alternative ones or, in this case, to new categories of responses. This finding indicates that self-efficacy is involved with cognitive flexibility, which subsequently engenders creativity.

Furthermore, positive affect partially mediated the relationship between creative self-efficacy and flexibility. This result suggests that people who are more confident in their creative ability experience more positive affect, and this positive affect is partially responsible for greater flexible thinking. High self-efficacy promotes positive affect (Linnenbrink and Pintrich, 2003), which in turn facilitates cognitive flexibility. Activating positive affect encourages people to explore new possibilities freely and flexibly by making them feel safe and free of problems; positive affect is also involved in the release of dopamine in certain brain areas that are related to cognitive flexibility (Nijstad et al., 2010). This cognitive flexibility subsequently enhances creativity.

Study 2 investigated creative convergent thinking measured by insight problems. Results from this study revealed that the creative growth mindset was positively related to task enjoyment, effort, and positive affect, whereas the fixed mindset was negatively related to task enjoyment and effort but positively related to negative affect. These results indicate that when performing a creativity task, people who firmly believe creativity is developable are likely to experience more positive affect, enjoy the task more, and exert more effort. On the other hand, the more people see creativity as an unchangeable ability, the more they experience negative affect, the less they find the task enjoyable, and the less they expend effort on it. In addition, a negative association was discovered between the fixed mindset and the number of solved insight problems. This result is in alignment with Karwowski’s (2014) finding and indicates that viewing creativity as undevelopable suppresses the effectiveness of problem-solving. The direct effects of creative self-efficacy and indirect effects of the beliefs on creativity failed to emerge.

The negative predictive effect of the fixed mindset on problem-solving may be explained by the inability of those who hold a stronger fixed belief to adapt when necessary. When solving a problem, the solver tends to explore the solution based on his or her experience first, and when that experience is insufficient to solve the problem, the solver steps into a state where he or she does not know what to do next (Knoblich et al., 1999). The solver must overcome the familiar way of thinking and come up with a new approach in order to find the solution (Dow and Mayer, 2004). Schroder et al. (2014) examined how induced mindsets influence cognitive control brain activity. They found that attention allocation to responses was enhanced immediately after exposure to a fixed mindset, but this attention was not related to behavioral change following errors, indicating that enhanced attention to responses does not lead to adaptive performance adjustments in people with a fixed mindset. As such, the limited ability to adjust observed among people who endorse the creative fixed mindset may lead to ineffectiveness in changing their way of thinking, resulting in unsuccessful problem-solving.

When comparing the results of the two studies, some discrepancies were observed. In Study 1, the predictive effects on the self-regulatory responses (i.e., affect, task enjoyment, and effort) and creativity (i.e., flexibility and originality scores) mostly emerged from creative self-efficacy, whereas the fixed and growth mindsets only affected affect. In Study 2, the predictive effects of self-efficacy were not detected at all, but more effects of creative mindsets were detected. Specifically, both mindsets predicted affect, task enjoyment, and effort, albeit in opposite directions. Additionally, the fixed mind set also predicted creative problem-solving. The differences could be due to the distinct nature of the tasks used to test creativity; that is, Study 1 employed the Alternative Uses Task as a measure of divergent thinking, while Study 2 used insight problems to assess convergent thinking. The associations between the beliefs and creativity may vary depending on tasks. Because the relationship between self-efficacy and performance is reciprocal (Bandura, 1989; Williams and Williams, 2010), it is possible that engaging in a difficult task lowers people’s confidence in their ability, thus weakening the effect of self-efficacy that was tested prior the creativity tasks. The insight problem-solving task used in Study 2 is more difficult than the Alternative Uses Task used in Study 1, hence the discrepancies in the results.

The general results of these two studies reveal similar trends in which creative self-efficacy and the creative growth mindset are linked to desirable outcomes. The fixed mindset, on the other hand, is associated with adverse results.

Several limitations of this research must be addressed. First, this research was cross-sectional and correlational in design. As a result, no claim can be made with respect to the causality of the relationships among variables. Second, the questionnaires employed in this research were translated from English to Thai. Although the scale reliabilities appeared to be adequate after the removal of some items, further studies are necessary to assess the validation of the scales used in this particular sample. Lastly, although the sample size for each study met the common minimum requirement of 200 cases for SEM studies (Kline, 2016), the models were quite complex, and thus larger sample sizes are recommended for future research.

Many questions concerning the effects of beliefs about creativity on creativity remain unanswered. First of all, there were some discrepancies between the results gathered using a divergent thinking task in Study 1 and those acquired using a convergent thinking task in Study 2. These discrepancies could exist because the associations between the beliefs and creativity vary depending on the task. Future research could explore the effects of the beliefs on various task types. Additionally, researchers could examine the impact of task difficulty. Given that the self-regulatory benefits of self-efficacy (Bandura, 1977) and incremental beliefs (Dweck, 2000) seem to be most apparent when people encounter obstacles, more effects of the beliefs on psychological outcomes and creativity might emerge or disappear when taking into account the level of task difficulty. Secondly, the present research only provided correlational results. In the future, longitudinal and experimental research should be conducted in order to confirm the directionality of the relationships among the beliefs, self-regulatory responses, and creativity. Finally, these findings only demonstrated the mediation effects of positive affect and flexibility on the relationship between creative self-efficacy and creative performance. Future research could replicate these results by testing the same variables used in this research and expanding the investigation to include further relevant factors.

## Conclusion

This research explores factors that could explain the relationship between beliefs about creativity (i.e., creative self-efficacy and mindsets) and creative performance. This research contributes additional knowledge regarding how beliefs concerning creativity, particularly creative self-efficacy beliefs, might influence creativity. The present findings suggest that creative self-efficacy could positively affect creativity by promoting positive affect and enhancing cognitive flexibility. This research also reveals some connections between beliefs concerning creativity and adaptive self-regulatory outcomes (i.e., affect, task enjoyment, and effort).

## Author Contributions

NH and NI designed this study. NI collected and analyzed the data. NI and NH wrote the article.

## Conflict of Interest Statement

The authors declare that the research was conducted in the absence of any commercial or financial relationships that could be construed as a potential conflict of interest.
